# Dihydrochalcone Compounds Isolated from Crabapple Leaves Showed Anticancer Effects on Human Cancer Cell Lines

**DOI:** 10.3390/molecules201219754

**Published:** 2015-11-27

**Authors:** Xiaoxiao Qin, Yun Feng Xing, Zhiqin Zhou, Yuncong Yao

**Affiliations:** 1 College of Horticulture and Landscape Architecture, Southwest University, Chongqing 400716, China; vipqindada@163.com; 2 Beijing Key Laboratory for Agricultural Application and New Technique, Department of Plant Science and Technology, Beijing University of Agriculture, Beijing 102206, China; 13521981395@163.com

**Keywords:** *Malus* crabapples, leaves, dihydrochalcone compounds, anticancer activity, human cancer cell lines

## Abstract

Seven dihydrochalcone compounds were isolated from the leaves of *Malus* crabapples, cv. “Radiant”, and their chemical structures were elucidated by UV, IR, ESI-MS, ^1^H-NMR and ^13^C-NMR analyses. These compounds, which include trilobatin (**A1**), phloretin (**A2**), 3-hydroxyphloretin (**A3**), phloretin rutinoside (**A4**), phlorizin (**A5**), 6′′-*O*-coumaroyl-4′-*O*-glucopyranosylphloretin (**A6**), and 3′′′-methoxy-6′′-*O*-feruloy-4′-*O*-glucopyranosyl-phloretin (**A7**), all belong to the phloretin class and its derivatives. Compounds **A6** and **A7** are two new rare dihydrochalcone compounds. The results of a MTT cancer cell growth inhibition assay demonstrated that phloretin and these derivatives showed significant positive anticancer activities against several human cancer cell lines, including the A549 human lung cancer cell line, Bel 7402 liver cancer cell line**,** HepG2 human ileocecal cancer cell line, and HT-29 human colon cancer cell line. **A7** had significant effects on all cancer cell lines, suggesting potential applications for phloretin and its derivatives. Adding a methoxyl group to phloretin dramatically increases phloretin’s anticancer activity.

## 1. Introduction

Dihydrochalcones are a class of flavonoids characterized by a basic C6-C3-C6 backbone structure and the absence of a heterocyclic C ring. Dihydrochalcones are considered to be the primary precursors and represent important intermediates in the synthesis of flavonoids [1,2]. Dihydrochalcones (mainly phloridzin, sieboldin, trilobatin, and phloretin) represent the major flavonoid subgroup in plant tissues [3]. They are widely distributed in apple trees, especially in the leaves and immature fruits. Recently, dihydrochalcones have attracted increasing interest due to their bioactivities. For example, phloridzin, the most abundant phenolic compound in *Malus* plants [2,4,5], regulates apoptosis and alters gene expression to inhibit the growth of cancer cells. It reduces the blood glucoside levels of normal BALB/c mice feed with a phloridzin-containing diet [6,7]. Bissinger reported that phloretin isolated from apples stimulates erythrocyte cell membrane scrambling and has effects in geriatric patients [8]. Moreover, phloretin and phloridzin have been reported to have antioxidant, anti-aging and anti-inflammatory activities [9,10,11]. Interestingly, most dihydrochalcones are natural sweetening agents. However, known dihydrochalcones from natural sources are rare, and the anticancer effects of dihydrochalcones have not been previously reported.

Crabapples belong to the genus *Malus* (Rosaceae). They are important ornamental plants widely distributed throughout the world. Although crabapples are mainly used for ornamental purposes, they also have other applications. The leaves of some cultivars, such as *Malus hupehensis* (Pamp.) Rehd are often used as a tea [12], and the fruits are consumed and utilized in the production of fruit beverages due to the fact they are rich in antioxidant flavonoids [13,14]. The medicinal value and antioxidant activity of crabapples have been reported in previous studies [14,15,16]. However, the dihydrochalcone components of crabapples are not well characterized. Our preliminary studies found that the number of types and contents of dihydrochalcone compounds in “Radiant” variety crabapple leaves are higher compared to those in *M. domestica* apple leaves (unpublished results).

Therefore, in this paper, we describe the isolation and structure of dihydrochalcone monomers from the *Malus* cultivar “Radiant” and provide evidence for the anticancer activities of these compounds, suggesting further investigations of their potential anti-tumor activities in animal models.

## 2. Results and Discussion

### 2.1. Separation and Purification of Dihydrochalcone Monomers in Malus Crabapples var. “Radiant”

**Figure 1:**
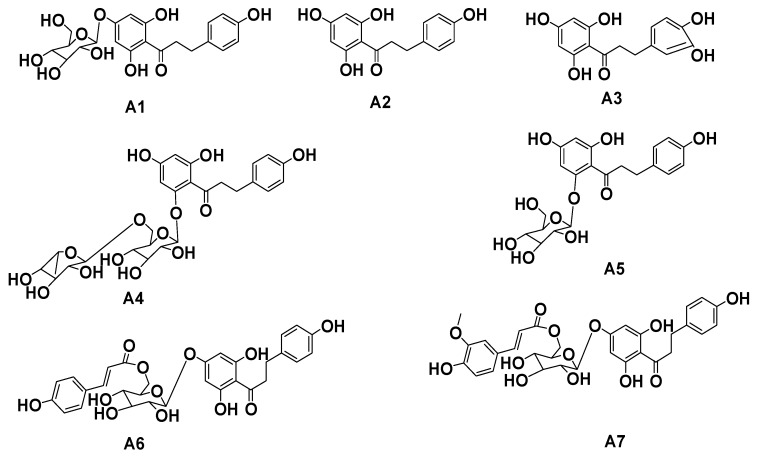
Structure of dihydrochalcone monomer compounds **A1**–**A7**.

The leaves of *Malus* crabapples were extracted with 50% ethanol/water. Evaporation of the solvent extract was carried out under reduced pressure. The extract was then subjected to column chromatography (CC) and preparative HPLC (PHPLC). Seven dihydrochalcones were identified in the extract, including two new rare dihydrochalcone compounds. These compounds are trilobatin (**A1**), phloretin (**A2**), 3-hydroxyphloretin (**A3**), phloretin rutinoside (**A4**), phlorizin (**A5**), 6′′-*O*-coumaroyl-4′-*O*-glucopyranosylphloretin (**A6**), 3′′′-methoxy-6′′-*O*-feruloy-4′-*O*-glucopyranosyl-phloretin (**A7**) (Figure 1).

The dihydrochalcone compound **A1** was isolated as a yellow powder, and the NMR analysis (500 MHz, CD_3_OD) yielded the following data: ^1^H-NMR δ: 7.03 (2H, d, *J* = 10 Hz, H-2, 6), 6.68 (2H, d, *J* = 10 Hz, H-3, 5), 6.09 (2H, brs, H-3′, 5′), 3.92 (1H, dd, *J* = 2.5 Hz, 15 Hz, H-α), 3.71 (1H, dd, *J* = 6 Hz, 15 Hz, H-α), 2.86 (2H, t, *J* = 10.5 Hz, H-β), and 4.93 (1H, d, *J* = 9.0 Hz, glc-H-1). The detailed ^13^C-NMR data are listed in Table 1. The above information was consistent with a previous report [17], and this compound was thus identified as trilobatin.

The dihydrochalcone compound **A2** was isolated as a colorless powder, and the ^1^H-NMR analysis yielded the following data: δ: 2.86 (2H, t, *J =* 7.6 Hz, H-β), 3.20 (2H, t, *J* = 7.6 Hz, H-α), 5.80 (2H, s, H-3′, 5′), 6.65 (2H, d, *J* = 8.5 Hz, H-3, 5), and 7.00 (2H, d, *J* = 8.5 Hz, H-2, 6). The detailed ^13^C-NMR data are listed in Table 1. The above information is consistent with a previous report [18] and this compound was thus identified as phloretin.

**Table 1:** ^13^C-NMR Data (125 MHz) of compounds **A1**, **A2**, **A3**, **A4** and **A5** in CD_3_OD.

C	A1	A2	A3	A4	A5
1	133.82	132.6	133.43	132.51	132.47
2	130.3	128.93	114.89	129.04	128.99
3	116.1	114.7	143.9	114.71	114.68
4	156.43	155.03	144.69	154.93	154.97
5	116.1	114.7	115.16	114.71	114.68
6	130.3	128.93	119.24	139.04	128.99
1′	106.87	103.91	103.91	105.5	105.37
2′	165.32	164.74	164.44	166.12	166.17
3′	96.41	94.34	94.33	97.04	96.93
4′	164.96	164.44	164.72	164.44	164.51
5′	96.41	94.34	94.33	94.24	94.01
6′	165.32	164.74	164.44	160.84	160.91
α	47.49	45.93	45.85	45.54	45.58
β	31.79	30.09	30.23	29.46	29.43
C=O	207.01	205	205.03	205.2	205.15
Sugar-1′′	101.49			100.87	100.66
2′′	74.61			75.79	73.3
3′′	78.24			78.08	77.08
4′′	71.13			70.95	69.67
5′′	77.88			73.32	77.01
6′′	62.36			66.28	61.01
1′′′				100.61	
2′′′				77.06	
3′′′				70.62	
4′′′				72.64	
5′′′				69.78	
CH_3_				16.52	

The dihydrochalcone compound **A3** was isolated as a yellow powder, and the ^1^H-NMR analysis yielded the following data: δ 6.69 (1H, d, *J =* 5 Hz, H-2), 6.68 (1H, d, *J =* 5 Hz, H-5), 6.56 (1H, dd, *J =* 10 Hz, 5 Hz, H-6), and 5.83 (2H, s, H-3′, 5′). The detailed ^13^C-NMR data are listed in Table 1. The above information was consistent with a previous report [19], so this compound was identified as 3-hydroxyphloretin.

The dihydrochalcone compound **A4** was isolated as a yellow powder, and the ^1^H-NMR analysis yielded the following data: δ 7.09 (2H, d, *J* = 10 Hz, H-2, 6), 6.71 (2H, d, *J* = 10 Hz, H-3, 5), 6.0 (1H, s, H-3′), 6.18 (1H, s, H-5′), 5.04 (1H, d, *J* = 5 Hz, glc-H-1), 4.73 (1H, brs, rha-H-1), and 1.22 (3H, d, *J* = 5 Hz, H-rha-Me). The detailed ^13^C-NMR data are listed in Table 1. These data were consistent with the spectral data of a compound found in *Combretum griffithii* [20], so this compound was identified as phloretin rutinoside.

The dihydrochalcone compound **A5** was isolated as a yellow powder, and the ^1^H-NMR analysis yielded the following data: δ 3.92 (2H, t, *J* = 12.1 Hz, H-α), 2.90 (2H, t, *J* = 7.7 Hz, H-β), 7.08 (2H, d, *J* = 8.5 Hz, H-2, 6), 6.70 (2H, d, *J* = 10 Hz, H-3, 5), 5.98 (1H, d, *J* = 2.3 Hz, H-3′), 6.20 (1H, d, *J* = 2.2 Hz, H-5′), and 5.06 (1H, d, *J* = 7.25 Hz, glc-H-1). The detailed ^13^C-NMR data are listed in Table 1. Similar results were reported for a compound isolated from strawberries [21], and this compound was thus identified as phlorizin.

**Figure 2:**
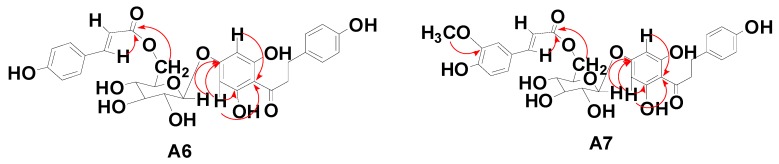
Main ^1^H-^1^H COSY and HMBC correlations of **A6** and **A7**.

The dihydrochalcone compound **A6** was isolated as a white powder. Its properties were characterized by negative-ion HRESI-TOF-MS (*m/z* 581.1641 [M − H]^−^, calcd for C_30_H_30_O_12_ 582.1737). The UV spectrum of **A6** showed the characteristic absorption peak of a dihydrochalcone, with a λ_max_ (MeOH) of 280 nm. The ^1^H-NMR spectrum (500 MHz, CD_3_OD) yielded the following data: δ 6.92 (2H, d, *J* = 10.5 Hz, H-2, 6), 6.68 (2H, d, *J* = 10.5 Hz, H-3, 5), 6.0 (2H, s, H-3′, 5′), 3.27 (1H, m, H-α), 3.37 (1H, m, H-α) and 2.82 (2H, m, H-β) [22], and contained all the phloretin signals. Moreover, the ^1^H-NMR spectrum also contained a glucose aglycone, δ 4.88 (1H, d, *J* = 9 Hz, glc-1). In addition to the above signals, the ^1^H-NMR spectrum showed a group of coumaric acyl signals of 6.59 (2H, d, *J* = 10 Hz, H-2′′′, 6′′′), 7.31 (2H, d, *J* = 10.5 Hz, H-3′′′, 5′′′), 6.26 (1H, d, *J* = 20 Hz, H-7′′′), and 7.47 (1H, d, *J* = 19.5 Hz, H-8′′′) [23]. The ^13^C-NMR (125 MHz, CD_3_OD) spectrum showed phloretin signals: δ 206.94 (C=O), 47.43 (C-α), and 31.05 (C-β), and a group of typical coumaric acyl signal and two double bond signals: δ 116.69 (C-7′′′), 146.85 (C-8′′′), and a carbonyl carbon signal 169.11 (C-9′′′). In addition, the carbon spectrum showed a glucoside signal. Finally, in the HMBC experiment (refer to **A6** in Figure 2), the end H of the glucoside δ 4.88, H-3′, 5′ (δ 6.0) was correlated with C-4′ (d, 164.59), and the H-3′ and H-5′ chemical shifts were the same in the H spectrum. These data show that the H-3′ and H-5′ are symmetric; thus, it could be inferred that the glucose is connected to the phloretin 4′-position. The visible H-7′′′ (δ 6.26) and glu-6′ (δ 4.46, 4.20) are all related to the coumaric acyl-C-9 (δ 169.11), indicating that the coumaric acyl group is connected to glu-6. The detailed ^13^C-NMR data are listed in Table 2. This compound was thus identified as 6′′-*O*-coumaroyl-4′-*O*-glucopyranosylphloretin.

The dihydrochalcone compound **A7** was isolated as a white powder. It was characterized by negative-ion HRESI-TOF-MS (*m/z* 611.1713 [M − H]^−^, calcd for C_31_H_32_O_13_ 612.1842). The UV spectrum of **A6** showed the characteristic dihydrochalcone absorption peak, with a λ_max_ (MeOH) of 280 nm. The ^1^H-NMR spectrum (500 MHz, CD_3_OD) contained all the phloretin signals, and yielded the following data: δ 7.03 (2H, d, *J* = 10 Hz, H-2, 6), 6.70 (2H, d, *J* = 10 Hz, H-3, 5), 6.12 (2H, s, H-3′, 5′), 3.27 (1H, m, H-α), 3.37 (1H, m, H-α) and 2.82 (2H, m, H-β) [22]. Moreover, the ^1^H-NMR spectrum also contained one methoxy δ 3.89 (3H, s) signal and one end of a glucose aglycone δ 4.97 (1H, d, *J =* 9 Hz, glc-1). In addition to the above signals, the ^1^H-NMR spectrum showed a group of feruloylated signals at 7.16 (1H, d, *J =* 2 Hz, H-2′′′), 7.05 (1H, d, *J =* 10 Hz, H-5′′′), 6.81 (1H, d, *J =* 10 Hz, H-6′′′), 6.43 (1H, d, *J =* 10 Hz, H-7′′′), and 7.60 (1H, d, *J =* 10 Hz, H-8′′′) [24]. The ^13^C-NMR (125 MHz, CD_3_OD) spectrum showed a set of phloretin dihydrochalcone signals: δ 205.61 (C=O), 46.12 (C-α), and 29.70 (C-β) The ^1^C-NMR spectrum also showed a group of typical feruloylated signals and two double bond signals: δ 113.70 (C-7′′′), 145.73 (C-8′′′), and carbonyl carbon signal at 169.70 (C-9′′′). In addition, the carbon spectrum contains a glucoside signal and one methoxy carbon at δ 55.04. Finally, in the HMBC experiment (**A7** in Figure 2), the end H of the glucoside δ 4.97, H-3′, 5′ (δ 6.12) was correlated with C-4′ (d163.92), and the H-3′ and H-5' chemical shifts were the same as in the H spectrum. These data showed that the H-3′ and H-5′ were symmetrical, which indicated that the glucose is connected to the phloretin 4'-position; the visible H-7′′′ (δ 6.43) and glu-6′ (δ 4.59, 4.30) are all connected to feruloylated-C-9 (δ 167.70), indicating that the feruloylate moiety is correlated to glu-6. Moreover, according to the HMBC spectrum, the methoxy is connected to the feruloyl C-3. The detailed ^13^C-NMR data are listed in Table 2. This compound was thus identified as 3′′′-methoxy-6′′-*O*-feruloy-4′-*O*-glucopyranosyl-phloretin.

**Table 2:** ^13^C-NMR Data (125 MHz) of compounds **A6** and **A7** in CD_3_OD.

C	A6	A7
1	133.85	132.47
2	130.27	128.94
3	116.07	114.72
4	156.24	155.02
5	116.07	114.72
6	130.27	128.94
1′	106.87	105.52
2′	165.18	163.32
3′	96.47	95.14
4′	164.59	163.92
5′	96.47	95.14
6′	165.18	163.36
α	47.43	46.12
β	31.05	29.7
C=O	206.94	205.61
Glc-1′′	100.7	99.49
2′′	74.51	74.28
3′′	78.83	76.56
4′′	71.79	70.51
5′′	75.52	73.21
6′′	64.56	63.28
coumaroyl 1′′′	114.86	113.78
2′′′	127.15	126.35
3′′′	131.19	149.19
4′′′	161.03	147.89
5′′′	131.19	128.94
6′′′	127.15	114.99
7′′′	116.69	113.78
8′′′	146.85	145.73
9′′′	169.11	167.7

### 2.2. in Vitro Cytotoxicity of the Seven Dihydrochalcone Monomer Compounds 

**Figure 3:**
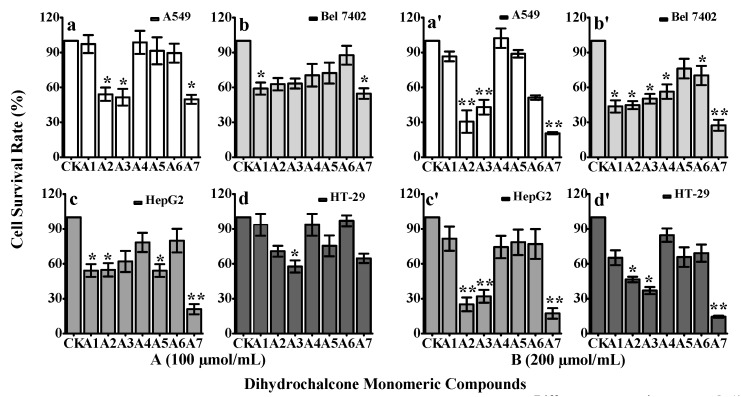
Anti-tumor therapeutic effects of the monomeric dihydrochalcone compounds from *Malus* “Radiant” crabapples. Human lung adenocarcinoma cell line A549, human hepatoma cell line Bel 7402, human cancer colorectal adenoma cell line HepG2 and colon cancer cell line HT-29 were treated with the indicated concentrations of various extracted fractions of *Malus* “Radiant” crabapples. The cell viability was then determined by an MTT assay, and the mean ± SD of six separate experiments was shown ((**A**) the concentration of monomeric compounds was 100 μmol/mL; (**B**) the concentration of monomeric compounds was 200 μmol/mL). The data shown are the mean ± SD. ** and * indicate significance at *p* < 0.01 and *p* < 0.05 by *t-*test, respectively.

The survival rates of tumor cells are shown in Figure 3. When the monomeric compounds **A1**–**A7** were administered at 100 μmol/mL, only **A2**, **A3**, **A6** and **A7** had any effect on A549 cells; **A1** and **A7** had effects on Bel 7402 cells; **A1**, **A2** and **A5** had some effects but **A7** dramatically inhibited the growth of HepG2 cells; and **A3** had effects on HT-29 cells (Figure 3A). When the concentration of monomeric compounds **A1**-**A7** was increased to 200 μmol/mL, **A2**, **A3**, **A6** and **A7** significantly inhibited the growth of A549 cells; **A1**-**A4**, **A6** and **A7** had effects on Bel 7402 cells; **A2**, **A3** and **A7** had significant effects on HepG2 cells; and **A2**, **A3** and **A7** had effects on HT-29 cells (Figure 3B). Collectively, except for compound **A5**, the other compounds showed significant cytotoxic activities against one or several of the four cancer cell lines. The two new, rare compounds **A6** and **A7** were more effective in targeting A549, Bel 7402, HepG2, and HT-29 cells. Methoxyl groups improve the antioxidant activity [25], and **A7** had significant effects on all tested cancer cell lines, perhaps due to its methoxyl group bonded to the phloretin structure that dramatically increases the anticancer activity of phloretin.

**Figure 4:**
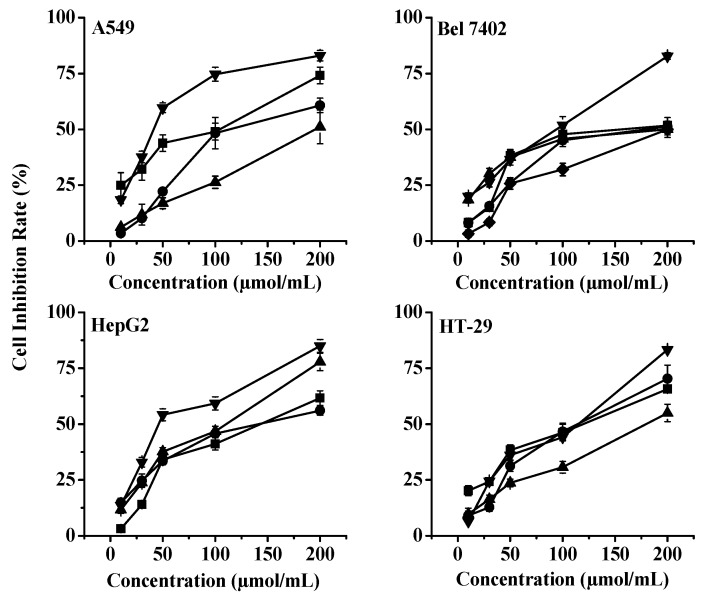
Inhibition rate of different monomeric compounds (A1 (◆), A2 (■), A3 (●), A6 (▲), A7 (▼)) in cancer cells. Cells (A549, Bel 7402, HepG2, HT-29) were treated for 24 h in the presence of the drug in medium. Cell viability was then determined by an MTT assay and is expressed as the mean ± SD of three separate experiments.

Because **A4** and **A5** had weaker anti-tumor effects than the other compounds, even under the highest concentration of 200 μmol/mL, we only used **A1**–**A3** and **A6**–**A7** to detect whether their anti-tumor effects depend on the concentration response (Figure 4). Notably, the results show that the concentration of monomeric compounds **A1**, **A2**, **A3**, **A6**, and **A7** had a positive correlation with the four tumor cell lines’ response curves. 

These data indicate that the cytotoxicity of dihydrochalcone monomer compounds (DMCs) on the four tumor cell lines are correlated with their bioactivity and was dose-dependent. Moreover, different tumor cells show different sensitivity to the inhibitory effects of the DMCs.

### 2.3. Cytotoxic Effects of Dihydrochalcone Monomeric Compounds on Four Human Cancer Cell Lines

After exposure to **A1**–**A7**, the IC_50_ values of the four cancer cell lines was determined and are shown in Table 3. Our data shows that different cells had different sensitivity to the inhibition effect of the dihydrochalcone monomers.

**Table 3:** IC_50_ (μg/mL) of various dihydrochalcones on cancer cells.

Compound	A549	BEL7402	HepG2	HT29
**A1**	>150	76.86 ± 4.97	>150	>150
**A2**	26.86 ± 3.11	37.08 ± 5.04	37.43 ± 1.23	33.20 ± 1.94
**A3**	39.83 ± 4.23	45.17 ± 8.02	37.79 ± 4.04	35.37 ± 2.53
**A4**	>150	>150	>150	>150
**A5**	>150	>150	>150	>150
**A6**	109.1 ± 18.31	86.61 ± 17.95	62.46 ± 7.20	99.87 ± 3.72
**A7**	39.79 ± 5.72	59.28 ± 5.06	49.36 ± 3.04	65.09 ± 2.77

The IC_50_ value reflects the intrinsic sensitivity of the acetylcholinesterase molecule to the inhibitor in rats [26]. In our experiments, the IC_50_ value can be understood as the concentration required to achieve 50% DMC-induced apoptosis of tumor cells. This concentration is known as the 50% inhibitory concentration. Namely, when the ratio between the apoptotic cells and all of the cells is greater than or equal to 50%, the IC_50_ is satisfied for every DMC concentration. Moreover, IC_50_ values can be used to measure the ability of DMC-induced apoptosis, and a stronger induction ability is associated with a lower IC_50_ value. The four tested cancer cell lines showed different sensitivities to the seven DMCs. For the A549 cells, the DMC induction ability order was **A2** > **A7** > **A3** > **A6** > **A1** ≈ **A4** ≈ **A5**; for Bel 7402 cells, the DMC induction ability was **A2** > **A3** > **A7** > **A1** > **A6** > **A4** ≈ **A5**; for HepG2 cells, the DMC induction ability was **A2** > **A3** > **A7** > **A6** > **A1** ≈ **A4** ≈ **A5**; and for HT-29 cells, the DMC induction ability was **A2** > **A3** > **A7** > **A6** > **A1** ≈ **A4** ≈ **A5**. The values of **A2**, **A3**, **A6** and **A7** indicate strong action against the four tumor cells. Although **A1** had strong anticancer activity for Bel 7402, its induction ability against A549, HepG2 and HT-29 cells was relatively weaker, and both **A4** and **A5** had weak induction ability against the four types of tumor cells.

Havsteen reported that the number and position of hydroxyl groups attached to the A-ring and the nature and position of the carbohydrate units in the glycosides could influence flavonoids’ medicinal uses [27]. Davide reported that the presence of a glycosyl moiety bound to the chalcone structure decreased the antimicrobial activity of phloretin [2]. These finding may explain why phloretin and its derivatives exhibited different induction abilities. In addition, phloretin (**A2**) showed the strongest induction ability among the derivatives: one explanation is its small molecular mass; the other is that some glycoside moiety is bound to the phloretin structure and decreases the anticancer activity of phloretin, such as is trilobatin (**A1**). Phlorizin (**A5**) has a glucoside, phloretin-rutinoside (**A4**) has a rutinoside, and 6′′-*O*-coumaroyl-4′-*O*-glucopyranosylphloretin (**A6**) and 3′′′-methoxy-6′′-*O*-feruloy-4′-*O*-glucopyranosylphloretin (**A7**) have a glucopyranosyl moiety.

## 3. Experimental Section 

### 3.1. Materials and Instruments

The *Malus* crabapples “Radiant” leaves used in the experiments were collected from 6-year-old *Malus* crabapple trees. Trees with similar growth vigor were planted in the Crabapple Germplasm Resource Garden of BUA (Beijing, China). When the leaves matured in mid-June, we collected 30 kg leaves for our experiments. Column chromatography with silica gel (#200–300 mesh), thin layer chromatography with silica gel GF254 (Qingdao Marine Chemical Plant, Qingdao, China); reverse phase silica gel C18 (including 50 m, YMC, Kyoto, Japan), Sephadex LH-20 (GE, Uppsala, Sweden); and MCI-gel CHP-20 p (70–150 μm, Mitsubishi Chemical Corporation, Tokyo, Japan). Analytically pure solvents were used. AB 8 macroporous resin (Hebei Bao An Resin Products Limited Liability Company, Baoan, China), Sephadex LH—20 from GE were used. The flavonoid composition of the crabapple leaves was detected by HPLC using an analytical column (Agilent Extend C18 4.6 × 250 mm) for HPLC analysis and a preparative chromatographic column (Agilent Extend, C18 9.4 × 250 mm) was used for isolating the monomeric compounds. NMR was performed with a Bruker DRX- 500 Nuclear Magnetic Resonance Instrument (Zug, Switzerland); Mass spectrometry analysis was performed with a Finnigan LCQ DECA XP PLUS Type Ion Trap Mass Spectrometer (San Jose, CA, USA) coupled to an Agilent 1260 HPLC (Palo Alto, CA, USA).

### 3.2. Purification of Dihydrochalcones from Crabapple “Radiant” Leaves

The air-dried crabapple leaves were powdered and extracted three times for 2 h with 70% MeOH at 60 °C. After the solvent evaporated, the pooled residues were suspended in water, and extracted sequentially with 30% MeOH, 60% MeOH on AB-8 macroporous resin. 

The 30% MeOH fraction was subjected to silica gel (200–300 mesh) column chromatography, with ethyl acetate:MeOH (1:0→50:1→20:1→3:1) gradient elution to give three fractions F1-F3. Among these fractions, F1 was subjected to Sephadex LH-20 column chromatography, eluted with MeOH, to give compound **A2**, which was further purified by preparative HPLC; F2 was eluted with chloroform:MeOH (10:1) on silica gel column (200–300 mesh), in a isocratic gradient manner, to afford **A4** that was purified by preparative HPLC. F3 was subjected to silica gel (200–300 mesh) column chromatography eluted in a isocratic gradient of chloroform–MeOH (10:1), to give two fractions F3_A_-F3_B_. F3_A_ was subjected to Sephadex LH-20 column chromatography, eluted with MeOH, to give compound **A3** that was purified by preparative HPLC.

The 60% MeOH fraction was subjected to silica gel (200–300 mesh) column chromatography, using gradient elution with petroleum ether-ethyl acetate (1:1→1:0), and ethyl acetate–MeOH (10:1→0:1), to obtain four fractions F4–F7. F4 was subjected to silica gel (200–300 mesh) column chromatography with gradient elution with chloroform–MeOH (10:1→5:1), to afford three fractions F4_A_-F4_C_; F4_B_ was subjected to Sephadex LH-20 column chromatography, eluted with MeOH, then, the separated compounds **A6** and **A7** were separately purified by preparative HPLC; F4_C_ was separated by Sephadex LH-20 column chromatography with MeOH as eluent and then purified by preparative HPLC to yield **A1**; F5 was subjected to silica gel (200–300 mesh) column chromatography, eluted in a stepwise gradient manner with chloroform–MeOH-water (8:1:0.05→5:1:0.05), to give two fractions F5_A_–F5_B_, then F5_B_ was subjected to Sephadex LH-20 column chromatography, eluted with MeOH. Finally, compound **A5** was purified by preparative HPLC with MeOH-0.01% TFA (60:40) and a 2.0 mL/min flow rate. Between each step, extracts were vacuum-filtered on a sintered glass funnel (porosity 4). Filtrates were subjected to evaporation, and the resulting powder was mixed with 20 g of silica. The soluble dihydrochalcones were eluted from the silica using CH_2_Cl_2_:MeOH (5:1).

### 3.3. MS System and Conditions

A Xevo G2-S QTof (Waters MS Technologies, Milford, MA, USA), a quadrupole and orthogonal acceleration time-of-flight tandem mass spectrometer, was used with an ESI source. Both positive and negative ion modes were used for compound ionization. The MSE data collection mode was used. At one sample injection, the mode could collect the exact mass data of the quasi-molecular ions and fragment ions by quickly alternating between low and high collision energies. The detecting conditions used were: capillary voltage of 0.45 kv, cone voltage of 40 v, source temperature of 120 °C, desolvation temperature of 500 °C, cone gas flow of 50 L/h, desolvation gas flow of 700 L/h, low energy of 6 V, and high energy ramp of 20 to 40 V. The Tof-MS mass range was *m/z* 100 to *m/z* 1200. The scan time was 0.2 s. All analyses were obtained using the Lockspray feature to ensure accuracy and reproducibility. The mass-to-charge ratio of leucine-enkephalin was used as the lockmass at a concentration of 200 ng/mL and a flow rate of 10 μL/min. The data were acquired by real time collection (scan time of 0.5 s, interval of 15 s). The UPLC-QTof-MS data of the samples were acquired and analyzed by the Waters UNIFI 1.7 software.

### 3.4. Cancer Cell Growth Inhibition

A MTT [3-(4,5)-dimethylthiazolyl)-3,5-diphenyltetrazolium bromide] cell viability assay was used. Succinate dehydrogenase in the mitochondria of live cells can convert the insoluble violet crystalline formazan produced from MTT and deposit it in the cells, but dead cells cannot not. Dimethyl sulfoxide (DMSO) could dissolve the formazan in cells, and its light absorption value was measured by ELISA at 490 nm. The number of living cells can be determined from the measured absorbance value (OD value).

Human lung adenocarcinoma cell line A549, human hepatoma cell line Bel7402, human cancer colorectal adenomas cell line HepG2 and colon cancer cell HT29 (ATCC, Manassas, VA, USA) were obtained from Dr. Wen Xu (College of Pharmacy, Fujian University of Traditional Chinese Medicine). These four tumor cell lines were picked in their logarithmic growth phase with trypsin digestion and cultured supplemented with 10% fetal bovine serum (GIBCO BRL, Carlsbad, CA, USA) in RPMI1640 Dulbecco's modified Eagle medium (DMEM) and reached a 150,00/mL cell suspension. Then, these cells were seeded into 96-well plates at 190 μL/plate at 37 °C for 24 h under 5% CO_2_. An aliquot of 10μL of the test compounds was added to cells and cultured at 37 °C for 3 days under 5% CO_2_. The cells were measured by a modified MTT assay [28,29]. The A549, Bel7402, HepG2 cells were treated with MTT solution (final concentration of 0.5 mg/mL in DMEM) for 4 h at 37 °C in a 96-well plate, the supernatant was carefully removed, and DMSO (150 μL) was added to each well to dissolve the precipitate. The absorbance at 570 nm was measured using a Model 680 microplate reader (BIO-RAD, Hercules, CA, USA).

### 3.5. Statistical Analysis

Data are presented as the mean ± standard deviation (SD). Analysis of variance (ANOVA, SPSS 17.0 software, SPSS Inc., Chicago, IL, USA) of all values was used to assess differences in the means among different samples (*p* < 0.05). Duncan’s multiple analysis and Student’s *t*-test were used to identify significant differences among groups (*p* < 0.05, *p* < 0.01). Graphs were prepared in Origin Pro 8.0 SR4 (Origin Lab, Northampton, MA, USA) and Adobe Photoshop CS6.

## 4. Conclusions

Seven dihydrochalcone compounds separated and purified from *Malus* crabapple var. “Radiant” leaves displayed remarkable biological activities. Compounds **A2**, **A3**, **A6** and **A7** had a strong protective effect against the four tested tumor cell lines A549, Bel 7402, HepG2 and HT-29. Therefore, we propose that the dihydrochalcones may have beneficial effects on human health and can be considered as possible therapeutic agents against cancer. This article shows that *Malus* crabapples may be a valuable resource of anticancer effects, and the two new rare compounds **A6** (6′′-*O*-coumaroyl-4′-*O*-glucopyranosylphloretin) and **A7** (3′′′-methoxy-6′′-*O*-feruloyl-4′-*O*-gluco-pyranosylphloretin) could be developed into promising anticancer agents.
